# Supplementary material for: The genome sequence of a hoverfly, Sphaerophoria taeniata (Meigen, 1822)
Source: Wellcome Open Res. 2023 Oct 18;8:474. [Version 1] doi: 10.12688/wellcomeopenres.19921.1 (PMC10905010; doi:10.12688/wellcomeopenres.19921.1)
Supplement: Peer review history

# Reviewer report: Reviewer response for version 1

**DOI:** 10.21956/wellcomeopenres.22060.r72710

**Published:** 2024-02-27

**Authors:** Thayná Lopes

**Affiliations:** 1 Laboratório de Citogenética e Entomologia Molecular, Universidade Estadual de Londrina, Londrina, State of Paraná, Brazil

In this note, the authors present the genome sequence of a hoverfly, *Sphaerophoria taeniata *(Meigen, 1822).

 I believe that even though it is a data note, some details would be important to enrich this work.

- The summary, for example, is extremely concise. I believe it would be interesting to add more information about the species. A greater description of the species, its importance, and the relevance of generating data for this type of insect would add to the summary section.
- Overall, I would like to congratulate the authors. The quality of the data presented is very high and well validated.

Are sufficient details of methods and materials provided to allow replication by others?

Yes

Is the rationale for creating the dataset(s) clearly described?

Yes

Are the datasets clearly presented in a useable and accessible format?

Yes

Are the protocols appropriate and is the work technically sound?

Yes

Reviewer Expertise:

Bioinformatics (genomes, proteomes and transcriptomics) and molecular genetics of insects

I confirm that I have read this submission and believe that I have an appropriate level of expertise to confirm that it is of an acceptable scientific standard.

# Reviewer report: Reviewer response for version 1

**DOI:** 10.21956/wellcomeopenres.22060.r72715

**Published:** 2024-02-20

**Authors:** Edson Ishengoma

**Affiliations:** 1 University of Dar es Salaam, Dar es Salaam, Dar es Salaam, Tanzania

This data note contains the first report on the genome of a hoverfly, *Sphaerophoria taeniata*. I'm pleased with the rigor of the cutting-edge approaches employed in the assembly and quality control processes. The resulting genome will undoubtedly play an important role in our understanding of the biology, ecology, and evolution of hoverflies and other related species.

 I'm a little concerned about only utilising BRAKER2's default mode to create protein annotations from this assembly. There are numerous model arthropods with well-curated protein representations in Swiss-Prot and other similar databases. It could have been more powerful to reference BRAKER2 predictions against the curated protein collection available from different arthropods utilising MAKER or other annotation workflows to gain more accurate annotation of the hoverfly genome.

Are sufficient details of methods and materials provided to allow replication by others?

Yes

Is the rationale for creating the dataset(s) clearly described?

Yes

Are the datasets clearly presented in a useable and accessible format?

Yes

Are the protocols appropriate and is the work technically sound?

Yes

Reviewer Expertise:

Genomics; Bioinformatics

I confirm that I have read this submission and believe that I have an appropriate level of expertise to confirm that it is of an acceptable scientific standard.
